# Novel Grade Classification Tool with Lipidomics for Indica Rice Eating Quality Evaluation

**DOI:** 10.3390/foods12050944

**Published:** 2023-02-23

**Authors:** Luyao Zhao, Xiaoliang Duan, Hongbin Liu

**Affiliations:** 1 Academy of National Food and Strategic Reserves Administration, Beijing 100037, China; 2 China Animal Disease Control Center, Beijing 100020, China

**Keywords:** indica rice, grade classification, lipidomics, eating quality, model

## Abstract

The eating quality evaluation of rice is raising further concerns among researchers and consumers. This research is aimed to apply lipidomics in determining the distinction between different grades of indica rice and establishing effective models for rice quality evaluation. Herein, a high-throughput ultrahigh-performance liquid chromatography coupled with quadrupole time-of-flight (UPLC-QTOF/MS) method for comprehensive lipidomics profiling of rice was developed. Then, a total of 42 significantly different lipids among 3 sensory levels were identified and quantified for indica rice. The orthogonal partial least-squares discriminant analysis (OPLS-DA) models with the two sets of differential lipids showed clear distinction among three grades of indica rice. A correlation coefficient of 0.917 was obtained between the practical and model-predicted tasting scores of indica rice. Random forest (RF) results further verified the OPLS-DA model, and the accuracy of this method for grade prediction was 90.20%. Thus, this established approach was an efficient method for the eating grade prediction of indica rice.

## 1. Introduction

Rice is one of the staple food across the world [1]. With the tremendous development of society, rice eating quality attracts increasing attention among researchers and consumers [2]. Thus, the assessment of the eating quality of rice [3] is vital in variety cultivation and merchandise selection. Generally, rice palatability quality is identified by the standard sensory evaluation method in China, which is labor intensive and time-consuming [4], and the result is usually subjective due to the prejudice and alteration of the sensory system depending on the different daily psychological and physical states of human beings [5]. Hence, a novel high-throughput, sensitive, and comprehensive analytical tool was required.

Lipids are one of the three main compositions in rice [6,7] and play important roles in cell membrane components, energy storage, and signal transduction. Studies have shown that lipids affect the eating quality of rice [8,9], but the details remain unclear. Previous lipid research on rice eating quality were mainly fatty acid composition studied by gas chromatography/mass spectrometry (GC-MS) [10]. However, only limited lipids were studied before, and more detailed information on the lipid content and composition of rice relating to eating quality is still required. Comprehensive identification of lipids within biological systems, known as lipidomics, with tens of thousands of lipids, provides conceptions to lots of physiological activities [11,12] and diseases [13]. As for the sufficient resolution, sensitivity, mass accuracy, fragment ion scanning capability, and lipid profile information, ultrahigh-performance liquid chromatography coupled with quadrupole time-of-flight (UPLC-QTOF/MS) [14] has been applied for comprehensive lipidomics study in foods. Data processing is vital in lipidomics analysis, and useful information can be extracted by appropriate methods [15]; then, an effective prediction model is established. Many data processing ways such as principal component analysis (PCA) [16], linear discriminant analysis (LDA) [17], and orthogonal partial least-squares discriminant analysis (OPLS-DA) [18] have been utilized and reported in food analysis [19,20]. However, limited study on rice eating quality prediction by lipids has been reported.

In the present research, a high-throughput, high-sensitivity, and high-coverage UPLC-QTOF/MS method for comprehensive lipidomics profile analysis of rice was used. First, a comprehensive lipidomics study of the content and composition of rice was carried out by UPLC-QTOF/MS. Then, differential lipids were determined from three grades of indica rice. Finally, a novel lipid-based model was developed for predicting rice eating quality. This result provides details for which lipids are related to the eating quality of rice and serve as data for rice breeders and researchers to cultivate new rice varieties with improved eating quality.

## 2. Materials and Methods

### 2.1. Chemical and Reagents

Acetonitrile (ACN), isopropanol (IPA), methanol, and methyl tert-butyl ether were bought from Fisher (Chicago, IL, USA). Formic acid and ammonium acetate of LC-MS-grade were from CNW (CNW Technologies, Dusseldorf, Germany). Ultra-pure water was obtained from Milli-Q instrument (Milipore, Burlington, MA, USA).

The internal standards d7-monoglyceride (MG, 18:1), d7-diacylglycerol (DG, 15:0/18:1), d7-triacylglycerol (TG,15:0/18:1/15:0), d7-glycerophosphatidic acid (PA, 15:0/18:1), d7-glycerophosphatidylcholine (PC, 15:0/18:1), d7-glycerophosphatidylethanolamine (PE, 15:0/18:1), d7-glycerophosphatidylglycerol (PG, 15:0/18:1), d7-glycerophosphatidylinositol (PI, 15:0/18:1), d7-glycerophosphatidylserine (PS, 15:0/18:1), d7-ceramide (Cer, C15), and d9-sphingomyelin (SM, 18:1/18:1) were purchased from Avanti (Birmingham, AL, USA). Mixed reagents of all internal standards with final concentration of 100 μg/mL were prepared in methanol and preserved at −20°C in the freezer until analysis.

### 2.2. Sample Preparation and Extraction Procedure

Based on the variety information issued by Ministry of Agriculture and Rural Affairs of China from 2019 to 2020, collected rice varieties by planting area account for more than 70% of the promotion area in 10 provinces. Indica paddy rice samples were collected during harvest season in 2020. Further details related to field trial characteristics such as variety and geographical location can be found in Table 1. Paddy rice samples were shelled by JDMZ-100 huller (DFJH, Beijing, China). After that, part of the rice was milled by CT-293-CyclotecTM mill (Foss, Suzhou, China). The milled rice and powder was kept at −80 °C until analysis.

The extraction procedure refers to that in a previous study [21] with minor modifications. In short, 20 ± 0.01 mg rice powder was mixed with 200 μL methanol already containing lipid internal standards. The solution was mixed before and after addition of 540 μL MTBE for 30 s. Then, 360 μL ultrapure water was added and mixed for 30 s. After that, the tube was put at 4 °C for 10 min equilibration, then centrifuged at 4 °C with 15,000 rpm for 10 min. Extraction in two phases was transferred to new tubes separately. After evaporation, residue in the upper phase was redissolved in 1 mL solution (ACN/IPA/H_2_O = 65:30:5, *v*/*v*/*v*) for lipidomics study.

### 2.3. Sensory Test

Sensory test was conducted in compliance with the Chinese National Standard “Method for Sensory Evaluation of Paddy or Rice Cooking and Eating Quality” (GB/T 15682-2008) and “Rice” (GB/T 1354-2009). In short, rice was cooked in cooker with a 1.3 (*w*/*w*, water/milled rice) ratio after soaking for 30 min in water. Then, cooked rice was tested and scored by 20 skilled and qualified panelists. All evaluations were conducted in separate partitions with cooked rice served in random order. After that, average eating score of each sample was calculated and analyzed by SPSS 19.0 (International Business Machines Corporation, New York, NY, USA, USA). According to GB/T 1354–2009, rice with high (score ≥ 90), medium (score ≥ 80), and low (score ≥ 70) eating quality was evaluated and rated as grade high, medium, and low, respectively.

### 2.4. UPLC-QTOF/MS Analysis

For lipidomics, the UPLC I-Class Plus Instrument (Waters, Manchester, UK) was applied. A BEH C18 Column (130 A, 1.7 µm, 2.1 mm × 50 mm; Waters, Manchester, UK) was used. Separation was carried out with acetonitrile/ultra-pure water (phase A, 60/40, *v*/*v*) and isopropanol/acetonitrile (phase B, 90/10, *v*/*v*), both within 10 mM ammonium acetate and 0.1% formic acid for positive and negative ionization mode analysis. Following the LC analysis, lipids were instantly detected through a tandem QTOF Mass System (Waters, Manchester, UK). Both positive and negative ionization analysis were carried out with settings: capillary voltage, 2.0 kV for positive and 1.0 kV for negative ion mode; desolvation gas flow rate, 900 L/h; and detection range, 100–1500 *m*/*z*. Leu-enkephalin (0.2 µg/mL) with a fixed *m*/*z* of 556.2771 for positive mode and *m*/*z* for 554.2615 for negative mode was utilized as internal standard during the whole acquisition process.

To guarantee the instrument stability and data quality, quality control (QC) samples were prepared by equal pooling in all rice samples with internal standards added-in to monitor the quality and robustness of the data [22]. QC samples were engaged every 10 samples of rice through the entire analysis procedure.

### 2.5. Statistical Analysis

The raw data of UPLC-QTOF/MS was loaded on QI software (Waters, Manchester, UK) for analysis, which was carried out by noise setting, baseline correction, alignment, peak detection, and compound identification. Compound determination was performed by the in-house lipids and metabolites database. The accurate contents of lipids were analyzed through peak areas, fragment intensities, and stable-labeled internal standard compounds [23].

These processed data were then imported into EZinfo 3.0 software (Umetrics, Umeå, Sweden) for Student’s *t*-test, ANOVA analysis, and orthogonal partial least-squares discriminant analysis. OPLS-DA, with distinct predictive and orthogonal information indicating between and within group variance, has the ability to identify which variables include the class-separating information [24,25]. To reduce the data noise induced over-fitting, ANOVA was applied to identify the significantly different lipids among three groups with *p* value ≤ 0.05. For ANOVA analysis, after parameter setting, the data distribution and variance homogeneity check were automatically carried out through EZinfo 3.0 software, and then lipids with *p* value ≤ 0.05 were listed.

After being analyzed and filtered through ANOVA analysis with *p* value ≤ 0.05 and VIP ≥ 1, the filtered biomarkers were finally identified.

## 3. Results and Discussion

### 3.1. Rice Sensory Test Analysis

Among the 51 samples, the sensory testing scores of indica rice (IR) are listed in Table 1. In accordance with GB/T 15682-2008 and GB/T 1354-2009, out of 51 samples, 6, 38, and 7 were at grades high, medium, and low, respectively, and the top three varieties at the high level were Hengliangyou, Ezhong 5, and Guangliangx 2. Among all the samples, the medium eating levels gained more samples, and this result was in accordance with the current situation of paddy rice planting in China [26,27]. As for the same variety, such as Guangliangx 2 planted in different locations, rice gained multiple scores, even in different grades. Studies [28,29] have shown that environment and region affect rice quality, which may be the cause of the taste results in this study, but further studies still need to be developed to acquire a comprehensive explanation.

### 3.2. Identified Rice Lipids by UPLC-QTOF/MS

Rice samples (61 of IR) and 10 QC samples were detected through positive and negative modes. There was no significant variation of RT and *m*/*z* (CV < 19.8% on UPLC-QTOF/MS) for internal standards added in the lipid profiles of 10 QC samples [30]. Principal component analysis (PCA) indicated that QC samples were tightly clustered (Figure S1) under the whole MS collection procedure, indicating good stability and precision of the measurements through the total test duration [31].

For the positive mode (ESI^+^), DG, TG, PA, PG, and PI produced [M + NH4]^+^, and other lipids, such as PE, LPE, and PS, possessed primarily [M + H]^+^. PC, LPC, and SM generated [M + CH_3_COO]^−^, and other lipids produced [M − H]^−^ in negative ion mode [32]. In addition, PC and LPC formed fragment ions such as 184.07, while other lipids produced [M + H − NL]^+^ or [M + NH4 − NL]^+^ ions mainly (NL, neutral loss). Different lipid classes usually possess fixed NL [33], such as PA (NL, 115.01), PE (NL, 141.02), PG (NL, 189.03), PI (NL, 277.04), etc. As for detected ion message (MS1 and MS2) and matching scores (≥98.5) with the lipid maps library, the unknown lipids were characterized. The recoveries of the 12 internal standards for lipids are listed in Table S1, which are between 78.39% and 106.3% at different added concentrations, exhibiting approving and satisfying results.

**Figure 1:**
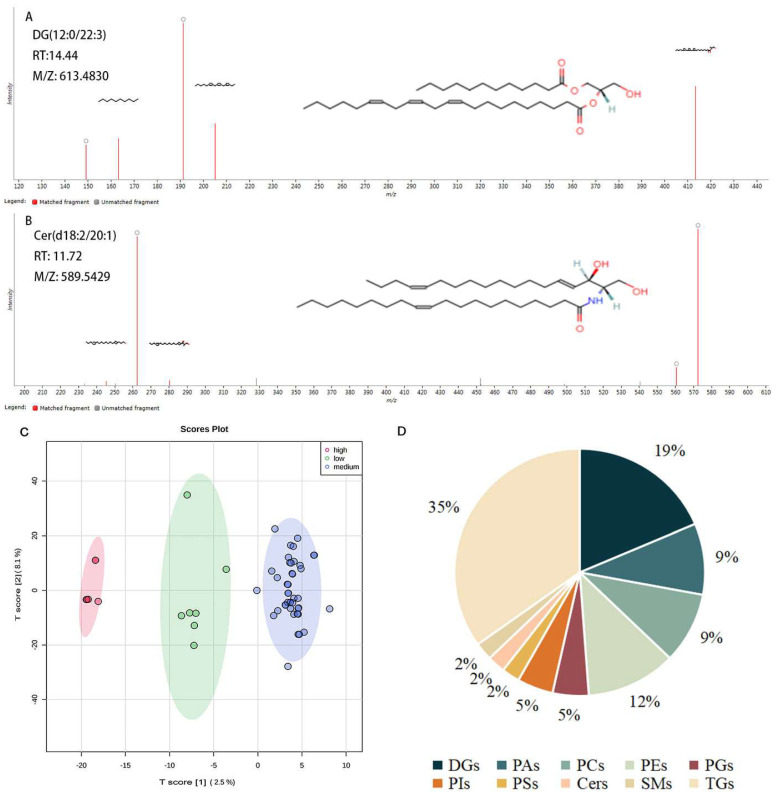
Lipid determination of rice. (**A**,**B**) Representative lipids of DG (12:0/22:3) and Cer (d18:2/20:1) after mapping with QI. (**C**) PCA plot of three grades of rice, with eating scores high (score ≥ 90), medium (score ≥ 80), and low (score ≥ 70). (**D**) Distribution of differential lipids of rice.

In the present study, the two representative matching images and compound structures from QI of DG (12:0/22:3) and Cer (d18:2/20:1) are shown in Figure 1A,B. PCA score plot of three grades of rice was presented in Figure 1C, a clear distinction among three grades could be seen basing on the lipids dataset. There were a total of 92 lipids identified in indica rice, including the 10 lipid classes DG, PA, PC, PE, PG, PI, PS, Cer, SM, and TG (Table S2). In Figure 1D, the % coverage of the pie plot is the percentage of each type of identified lipid number in the total identified lipids quantity of rice. Most identified lipids in rice were TGs and DGs, with proportions of 35% and 19% (Figure 1D), respectively, of total identified lipids.

### 3.3. Significantly Different Lipids among Three Eating Grades of Indica Rice

After filtering by *t*-test and ANOVA analysis, the details of potential lipid biomarkers with *p*-value ≤ 0.05 and VIP ≥ 1 are listed in Table 2, including category, retention time, and contents. In Table 2, forty-two lipids displayed significant difference of three sensory grades, containing eight DGs, four PAs, four PCs, five PEs, two PGs, two PIs, one PS, one Cer, one SM, and fourteen TGs. Lipids such as PC (18:1/18:2), PA (16:0), and PE (22:0) presented the highest contents in the high tasting value group compared to the medium and low ones and showed score dependence among three groups. The content of PC (18:1/18:2) ranged from 22.36 to 8.64 μg/kg, PA (16:0) from 171.41 to 136.59 μg/kg, and PE (22:0) from 100.15 to 44.41 μg/kg at high, medium and low sensory levels, respectively.

**Figure 2:**
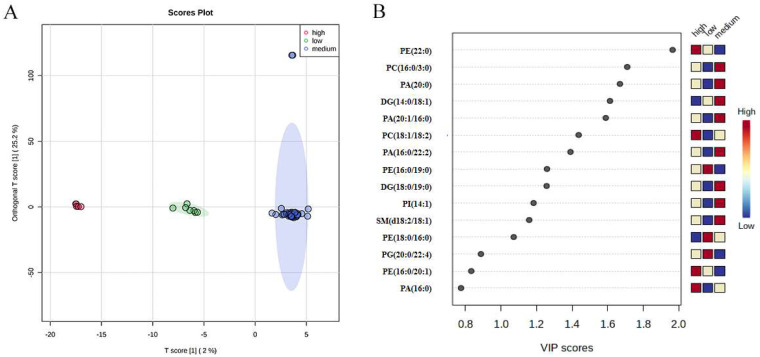
Significantly different lipids for *indica* rice. (**A**) OPLS-DA plot of lipid biomarkers for three grades of rice. (**B**) VIP scores for significantly different lipid species of OPLS-DA for three groups. Red and blue colors indicate high and low contents, respectively.

OPLS-DA was applied in view of lipids components to determine whether the three taste-level groups (high, medium, and low) of rice samples could be differentiated. Compared with PCA, OPLS-DA is a supervised method which obtains better classification results than PCA and results with reduced overfitting compared to PLS-DA. As shown in Figure 2A, an obvious distinction between the three groups could be seen in OPLS-DA. To further test the effectiveness of this OPLS-DA model, a permutation test (200 times) was carried out. Results show that this model was acceptable and valid with R2Y = 0.961 and Q2 = 0.928 (*p* < 0.005) [34]. In addition, OPLS-DA engaged VIP analysis to gain the most important lipids for the classification of the three groups. In the VIP analysis, the top 15 biomarkers with VIP values are presented in Figure 2B, and the first 3 VIP scores of lipids were 1.98, 1.69, and 1.67 for PE (22:0), PC (16:0/3:0), and PA (20:0), respectively. Glycerophospholipids, sphingolipids, and glycerolipids were highly responsible for the indica rice tasting scores. Since there were limited samples in each group, and sizes of the three groups were not inconsistent, the classification bias was inevitable. Thus, more analysis such as random forest analysis and correlation analysis should be performed to further test the OPLS-DA model. In addition, these differential lipids still need to be tested further with more samples in each group.

Fatty acids are one of the main class of lipids in rice; however, there were no significant differences in the high, medium, and low levels of indica rice in the present study. This result was in accordance with previous research [10]. In general, lipids in rice contains starch lipids and non-starch lipids, playing significant roles in the cooking and eating quality of rice. Lipids are usually bound to shape complexes with amylose and amylopectin, and in turn, influence the viscosity and gel consistency of the rice texture and elasticity. TG is one of the main lipid classes in rice, which is generally located in the rice body of bran and germ fractions as storage lipids in seeds [35]. DG, as the degradation product and also the precursor of TG, was a signal factor in many physiological activities. TG and DG are the main components of non-starch lipids and also present in tiny amounts in the starch lipids in rice [35]. In the present study, glycerophospholipids and glycerolipids gained more weight than other lipids in distinguishing the three taste groups for indica rice (Table 2, Figure 2C), indicating their importance in deciding rice quality.

PLs are fundamental proportions of cell membranes, including mitochondrial and endoplasmic reticulum [36]. In rice, PLs are more abundant in starch lipids than the non-starch of rice bran and germ, with PC, PE and PI serving as the principal PLs [37]. Sphingolipids, playing a vital role in signal transduction processes, are important components of biological biomembranes [38]. Studies have shown that starch lipids in rice have more effect on rice tasting quality than non-starch lipids; however, whether more or less lipid starch increases the quality is still controversial [10]. In addition, to our knowledge, there have been few targeted studies on detailed structures and compositions of these starch-lipids within rice grains, so more information needs to be investigated further.

### 3.4. Validation of Grade Classification Model for Indica Rice

**Figure 3:**
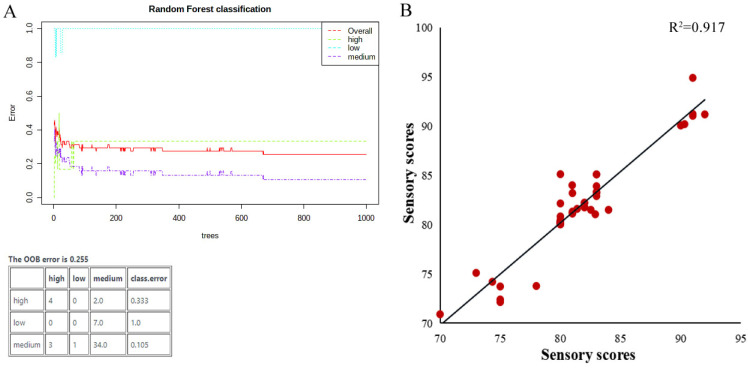
Grade-predicted model validation of rice. (**A**) RF results with lipids biomarkers. (**B**) Correlation study of sensory scores between actual and predicted ones by potential lipid biomarkers.

To assess the effectiveness of these lipid biomarkers for evaluating the grade level of indica rice further, random forest and correlation analysis were applied in this study. Random forest (RF) [39] is a classification method employing in lipidomics technology owing to the diverse rules of OPLS-DA. As presented in Figure 3A, the OOB error of the established model was only 0.255, and six samples were not classified correctly during RF analysis. RF data display the precision of this method for grade classification at 90.20%, indicating the accuracy of this model was greater than 90%.

During correlation analysis, sensory scores of rice were predicted by the lipid biomarkers model, and the scatter plot with practical and predicted sensory scores for indica rice is presented in Figure 3B. The correlation coefficient between the actual and predicted tasting scores was acquired for indica rice as 0.917. These results indicated this lipid-based model possesses high predictive ability and accuracy and could be used as a supplement for the current sensory evaluation of indica rice.

Screening the vital lipid points that related to rice tasting quality is necessary to ensure a desired method through biomarker-based models. Recently, researchers have focused on the impact of lipids on rice eating quality. Concepcion et al. [40] showed a clear distinction of lipid profiles between waxy and non-waxy rice. Researchers also studied the lipid components on rice cooking [41] and storage quality [9]. To our knowledge, there have been no studies concentrating on the lipids model by advanced UPLC-QTOF/MS for screening potential lipid biomarkers in identifying the taste quality of indica rice in China. In the present study, with the high-throughput information of lipid profiles and multivariate statistical analysis, obvious differentiation results (Figure 2 and Figure 3) showed lipids having exact relationships with indica rice eating quality and also presented which lipids (Table 2, Figure 2 and Figure 3) really affect it. However, more work is still required to assess the stability and effectiveness of this group of lipid biomarkers among other cultivars and different statuses of rice.

## 4. Conclusions

In this study, lipidomics was applied to identify the distinction of lipid composition at different grades (high, medium, and low) for indica rice. In total, 42 lipids displayed significant difference among 3 sensory grades, containing 8 DGs, 4 PAs, 4 PCs, 4 PEs, 2 PGs, 2 PIs, 1 PS, 1 Cer, 1 SM, and 14 TGs. A novel OPLS-DA model with this set of lipids for indica rice gradation was established. The RF result showed the accuracy of this model was greater than 90%. Thus, the developed lipid-based model could serve as a substitute tool for traditional sensory evaluation of indica rice in food and breeding departments.

## Figures and Tables

**Table 1:** Cultivation region, variety, grade, and average tasting scores with standard deviation (SD) of *indica* rice (IR) analyzed.

No.	Cultivation Regions	Varieties	Average Tasting Scores	Grade
1	Guilin, Guangxi	Yexiangyoulisi	80.54 ± 3.03	medium
2	Nanning, Guangxi	Yexiangyoulisi	82.53 ± 0.88	medium
3	Nanning, Guangxi	Guangliangx2	80.98 ± 5.32	medium
4	Yulin, Guangxi	Guangliangx2	70.36 ± 5.47	low
5	Nanning, Guangxi	Guangliangx2	90.32 ± 10.02	high
6	Guigang, Guangxi	Guangliangx2	81.03 ± 5.32	medium
7	Guigang, Guangxi	Guangliangx2	84.05 ± 10.09	medium
8	Nanning, Guangxi	Yexiangyoulisi	80.46 ± 7.60	medium
9	Laibin, Guangxi	Yexiangyoulisi	80.75 ± 3.11	medium
10	Guigang, Guangxi	Yexiangyoulisi	74.35 ± 6.04	low
11	Laibin, Guangxi	Yexiangyou2	80.03 ± 0.69	medium
12	Liuzhou, Guangxi	Yexiangyou2	83.77 ± 9.05	medium
13	Hezhou, Guangxi	Yexiangyou2	83.84 ± 10.77	medium
14	Nanning, Guangxi	Taiyou1002	70.13 ± 4.55	low
15	Jiangmen, Guangdong	Jinxiang	75.73 ± 4.64	low
16	Zhaoqing, Guangdong	ZayouH3	81.39 ± 2.76	medium
17	Zhaoqing, Guangdong	ZayouH3	80.30 ± 8.39	medium
18	Zhaoqing, Guangdong	ZayouH3	82.98 ± 1.24	medium
19	Yunfu, Guangdong	Changliangyou8	81.23 ± 4.82	medium
20	Shaoguan, Guangdong	ZayouH3	90.11 ± 6.23	high
21	Guangzhou, Guangdong	Changchun	82.28 ± 4.09	medium
22	Qingyuan, Guangdong	ZayouH3	83.37 ± 0.34	medium
23	Shaoguan, Guangdong	Hengliangyou	90.19 ± 2.40	high
24	Chenzhou, Hunan	Taiyou390	75.39 ± 5.61	low
25	Chenzhou, Hunan	Taiyou390	80.23 ± 5.14	medium
26	Chenzhou, Hunan	Taiyou390	83.28 ± 6.04	medium
27	Yueyang, Hunan	Zhaoyou5455	80.36 ± 0.36	medium
28	Yueyang, Hunan	Zhaoyou5455	80.09 ± 3.60	medium
29	Yueyang, Hunan	Zhaoyou5455	80.15 ± 0.99	medium
30	Yueyang, Hunan	Zhaoyou5455	83.20 ± 6.37	medium
31	Yiyang, Hunan	Huanghuazhan	75.28 ± 5.48	low
32	Yiyang, Hunan	Huanghuazhan	80.34 ± 0.95	medium
33	Yiyang, Hunan	Huanghuazhan	73.28 ± 6.54	low
34	Yiyang, Hunan	Huanghuazhan	81.73 ± 6.03	medium
35	Xiaogan, Hubei	Fengliangyou	80.52 ± 3.59	medium
36	Xiaogan, Hubei	Fengliangyou	80.36 ± 9.03	medium
37	Jingmen, Hubei	Ezhong5	90.84 ± 9.18	high
38	Jingmen, Hubei	Ezhong5	91.67 ± 6.20	high
39	Jingmen, Hubei	Jianzhen2	89.36 ± 2.78	medium
40	Jingmen, Hubei	Ezhong5	92.61 ± 2.03	high
41	Xiaogan, Hubei	Changliangyou8	80.95 ± 0.49	medium
42	Xiaogan, Hubei	Changliangyou8	81.36 ± 5.08	medium
43	Xiaogan, Hubei	Longliangyou534	83.48 ± 5.01	medium
44	Wuhu, Anhui	Fengliangyou	82.77 ± 1.39	medium
45	Wuhu, Anhui	Fengliangyou	82.64 ± 2.45	medium
46	Wuhu, Anhui	Fengliangyou	80.60 ± 0.44	medium
47	Wuhu, Anhui	Fengliangyou	80.37 ± 5.13	medium
48	Wuhu, Anhui	Fengliangyou	80.78 ± 6.24	medium
49	Chuzhou, Anhui	Fengliangyou	82.79 ± 5.10	medium
50	Chuzhou, Anhui	Huiliangyousm	80.37 ± 6.11	medium
51	Chuzhou, Anhui	Jingliangyouhz	83.92 ± 4.03	medium

**Table 2:** Annotations and contents (μg/kg) of significantly different lipids in three sensory grades (high, medium and low) of *indica* rice.

Category	Lipids	*m*/*z*	ESI Mode	Retention Time (min)	*p*-Value	Mean ± SD (High)	Mean ± SD (Medium)	Mean ± SD (Low)
DGs	DG(12:0/20:5)	603.4287	ESI pos	11.03	0.048	156.37 ± 3.64	104.83 ± 3.28	133.36 ± 11.24
	DG(14:0/18:1)	573.4874	ESI pos	14.36	0.020	1058.26 ± 14.11	1999.31 ± 19.70	1565.49 ± 22.11
	DG(16:0/17:1)	689.5330	ESI pos	19.15	0.008	0.74 ± 0.23	5.47 ± 0.16	16.49 ± 0.19
	DG(16:1/18:2)	608.5283	ESI pos	11.59	0.034	3.40 ± 0.57	11.23 ± 0.14	23.66 ± 0.58
	DG(17:2/20:5)	647.4589	ESI pos	13.19	0.016	33.43 ± 0.46	60.15 ± 0.81	86.25 ± 0.74
	DG(18:0/19:0)	603.5644	ESI pos	19.85	0.011	2725.25 ± 0.49	2897.24 ± 0.77	2627.08 ± 0.94
	DG(18:2/22:0)	721.5659	ESI pos	20.22	0.009	41.27 ± 0.06	76.08 ± 0.75	100.18 ± 0.13
	DG(18:3/18:1)	599.5038	ESI pos	11.56	0.014	32.09 ± 0.47	58.60 ± 0.15	101.95 ± 0.89
PAs	PA(20:1/16:0)	747.5158	ESI neg	13.79	0.035	471.67 ± 0.23	644.74 ± 0.07	374.55 ± 0.53
	PA(16:0)	417.2424	ESI neg	5.77	0.030	171.41 ± 7.15	142.79 ± 2.39	136.59 ± 1.73
	PA(16:0/22:2)	747.5549	ESI neg	11.15	0.025	6094.37 ± 100.33	7086.97 ± 71.90	5072.69 ± 32.77
	PA(20:0)	747.5095	ESI neg	13.78	0.024	7.03 ± 1.89	12.20 ± 1.49	0.15 ± 0.01
PCs	PC(18:1/18:2)	784.5857	ESI neg	11.00	0.028	22.36 ± 0.67	15.63 ± 0.36	8.64 ± 2.68
	PC(24:0/0:0)	606.4571	ESI neg	11.06	0.023	0.11 ± 0.00	0.29 ± 0.01	5.83 ± 0.05
	PC(14:0/18:3)	748.5089	ESI neg	12.65	0.028	2.36 ± 0.23	5.63 ± 0.95	18.64 ± 3.64
	PC(16:0/3:0)	536.3743	ESI neg	11.04	0.029	18.35 ± 2.28	27.99 ± 3.13	13.76 ± 3.78
PEs	PE(16:0/19:0)	759.6039	ESI pos	13.61	0.038	204.55 ± 10.81	193.15 ± 9.57	237.86 ± 13.88
	PE(18:0/16:0)	766.5954	ESI pos	10.55	0.001	3.61 ± 0.29	4.56 ± 0.99	5.66 ± 0.83
	PE(18:1/16:0)	718.5364	ESI pos	11.62	0.009	0.18 ± 0.04	0.01 ± 0.00	10.15 ± 0.19
	PE(16:0/20:1)	764.6152	ESI pos	11.54	0.003	5.56 ± 0.83	2.20 ± 0.12	3.86 ± 0.78
	PE(22:0)	502.3680	ESI pos	11.93	0.006	100.15 ± 10.01	61.81 ± 1.02	44.41 ± 0.03
PGs	PG(16:0/19:1)	747.5461	ESI neg	13.71	0.029	0.44 ± 0.01	9.72 ± 0.01	0.20 ± 0.00
	PG(20:0/22:4)	859.5766	ESI neg	12.60	0.019	2.26 ± 0.01	1.55 ± 0.21	6.12 ± 0.01
PIs	PI(14:1)	541.2464	ESI neg	10.21	0.007	2.53 ± 0.44	59.59 ± 13.04	1.57 ± 0.65
	PI(20:0/18:3)	897.5873	ESI neg	4.30	0.032	2.37 ± 0.40	1.53 ± 0.30	2.67 ± 0.05
PSs	PS(20:0/21:0)	884.6331	ESI neg	10.89	0.017	54.85 ± 1.48	50.16 ± 1.22	56.23 ± 0.07
Cers	Cer(d18:0/20:0)	596.5984	ESI neg	13.05	0.018	0.02 ± 0.00	1.47 ± 0.01	3.02 ± 0.14
SMs	SM(d18:2/18:1)	691.5624	ESI pos	6.42	0.025	1.06 ± 0.11	3.11 ± 0.31	0.15 ± 0.04
TGs	TG(14:0/16:0/20:0)	852.8019	ESI pos	20.39	0.026	136.90 ± 2.01	161.38 ± 2.75	144.72 ± 2.24
	TG(14:1/20:0/22:0)	958.8808	ESI pos	19.17	0.021	132.00 ± 2.35	299 ± 3.97	142 ± 7.09
	TG(15:0/18:1/20:1)	890.8183	ESI pos	20.15	0.006	554.54 ± 5.06	463.98 ± 9.90	585.02 ± 9.97
	TG(15:0/18:2/20:3)	884.7708	ESI pos	17.26	0.033	10.52 ± 0.33	12.01 ± 0.63	8.45 ± 0.53
	TG(16:0/18:1/20:0)	906.8496	ESI pos	20.74	0.009	89.72 ± 4.43	79.64 ± 2.12	82.36 ± 6.25
	TG(16:0/18:1/20:1)	904.8337	ESI pos	20.36	0.007	1001.31 ± 20.27	920.96 ± 13.43	1087.61 ± 14.91
	TG(16:1/18:2/20:3)	896.7713	ESI pos	16.71	0.009	300.09 ± 9.72	484.16 ± 2.53	333.33 ± 0.30
	TG(17:2/20:2/21:0)	956.8659	ESI pos	20.43	0.008	101.99 ± 3.39	196.47 ± 1.75	95.60 ± 4.55
	TG(17:2/20:2/22:0)	970.8810	ESI pos	20.64	0.007	6.62 ± 0.69	7.94 ± 0.65	7.06 ± 0.23
	TG(17:2/21:0/22:2)	984.8973	ESI pos	20.79	0.010	24.93 ± 1.67	18.68 ± 2.35	10.61 ± 2.98
	TG(18:1/20:0/22:2)	986.9131	ESI pos	21.06	0.010	8.86 ± 1.96	9.20 ± 0.96	10.87 ± 0.61
	TG(18:4/19:0/20:2)	962.8070	ESI pos	19.26	0.046	20.29 ± 1.44	48.06 ± 5.41	23.50 ± 2.64
	TG(18:4/19:0/22:0)	985.8877	ESI pos	17.90	0.027	5.27 ± 0.92	2.95 ± 0.58	2.61 ± 0.37
	TG(20:0/20:4/22:0)	959.8819	ESI pos	20.68	0.007	28.75 ± 0.25	58.85 ± 3.81	103.49 ± 8.51

## Data Availability

The data presented in this study are available on request from the corresponding author.

## Supplementary Materials

The following supporting information can be downloaded at: https://www.mdpi.com/article/10.3390/foods12050944/s1, Figure S1: The PCA score plots of compound abundances based on QC samples; Table S1: Recovery of different lipids at low, medium and high concentrations; Table S2: All lipids including ten categories identified in indica rice.
