# Supplementary material for: Novel Grade Classification Tool with Lipidomics for Indica Rice Eating Quality Evaluation
Source: Foods. 2023 Feb 23;12(5):944. doi: 10.3390/foods12050944 (PMC10000924; doi:10.3390/foods12050944)
Supplement: Supplementary file 1 [file foods-12-00944-s001.zip › foods-2088628-supplementary.pdf]

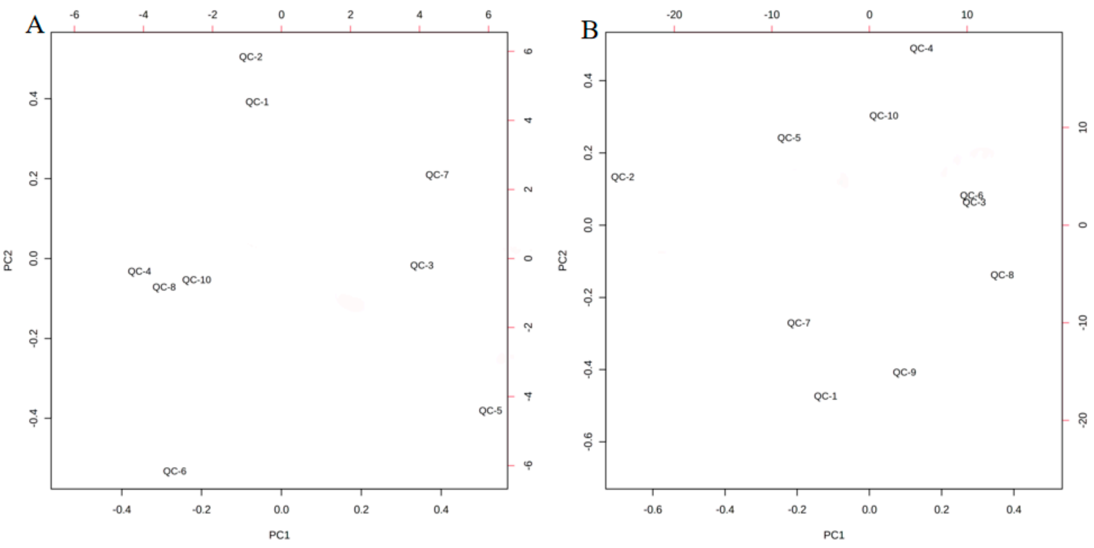

Figure S1: The PCA score plots of compound abundances based on QC samples. (A). PCA scores of lipidomics (pc1 from -0.37 to 0.56, and pc2 from -0.57 to 0.58). (B). PCA scores of metabolomics (pc1 from -0.64 to 0.40, and pc2 from -0.48 to 0.55).

Table S1: Recovery of different lipids at low, medium and high concentrations.

**Lipids**	**Exact mass**	**Recovery at low concentration (10ug/kg, %)**	**Recovery at medium concentration (100ug/kg, %)**	**Recovery at high concentration (1ug/g, %)**
15:0-18:1(d7) PE	710.5591	83.32	90.57	95.40
15:0-18:1(d7) PG	741.5537	78.39	82.14	86.23
15:0-18:1(d7) PI	829.5698	100.02	98.81	90.10
15:0-18:1(d7) PS	754.5690	82.35	89.56	83.04
C15 Ceramide-d7	530.5404	106.03	100.19	103.56
15:0-18:1(d7)-15:0 TG	811.7646	99.11	85.61	88.54
15:0-18:1(d7) DG	587.5506	98.66	90.34	88.28
18:1(d7) MG	363.3366	94.15	97.33	80.31
18:1(d7) LPE	486.3451	80.12	86.57	81.20
d18:1-18:1(d9) SM	738.6819	79.88	80.56	91.43
15:0-18:1(d7) PA	706.5158	80.23	98.75	94.73
15:0-18:1(d7) PC	753.6134	83.44	90.31	99.24

Table S2: All lipids including ten categories identified in *indica* rice.

Category	Lipids	m/z	ESI Mode	Retention time (min)
DGs	DG(12:0/20:5)	603.4287	ESI pos	11.03
	DG(14:0/18:1)	573.4874	ESI pos	14.36
	DG(16:0/17:1)	689.5330	ESI pos	19.15
	DG(16:1/18:2)	608.5283	ESI pos	11.59
	DG(17:2/20:5)	647.4589	ESI pos	13.19
	DG(18:0/19:0)	603.5644	ESI pos	19.85
	DG(18:2/22:0)	721.5659	ESI pos	20.22
	DG(18:2/24:0)	749.5632	ESI pos	20.26
	DG(18:3/18:0)	561.5032	ESI pos	11.58
	DG(18:3/18:1)	599.5038	ESI pos	11.56
	DG(21:0/22:5)	747.5702	ESI pos	13.58
	DG(20:1/18:1)	666.6031	ESI pos	20.01
	DG(24:0/18:2)	722.6657	ESI pos	23.49
PAs	PA(20:1/16:0)	747.5158	ESI neg	13.79
	PA(16:0)	417.2424	ESI neg	5.77
	PA(16:0/22:2)	747.5549	ESI neg	11.15
	PA(17:0)	431.5045	ESI neg	13.78
	PA(18:0)	445.5023	ESI neg	13.78
	PA(19:0)	459.5120	ESI neg	13.78
	PA(20:0)	747.5095	ESI neg	13.78
PCs	PC(18:1/18:2)	784.5857	ESI neg	11.00
	PC(24:0/0:0)	606.4571	ESI neg	11.06
	PC(14:0/18:1)	754.5102	ESI neg	12.21
	PC(14:0/18:2)	752.5102	ESI neg	12.41
	PC(14:0/18:3)	748.5089	ESI neg	12.65
	PC(16:0/3:0)	536.3743	ESI neg	11.04
	PC(18:1/18:1)	844.6073	ESI neg	15.31
	PC(18:1/18:2)	842.5917	ESI neg	14.89
	PC(18:2/18:2)	840.5760	ESI neg	13.42
PEs	PE(16:0/19:0)	759.6039	ESI pos	13.61
	PE(18:0/16:0)	766.5954	ESI pos	10.55
	PE(17:0/18:0)	759.6039	ESI pos	13.60
	PE(18:1/16:0)	718.5364	ESI pos	11.62
	PE(17:1/17:0)	718.5345	ESI pos	11.68
	PE(16:0/20:1)	764.6152	ESI pos	11.54
	PE(22:0)	502.3680	ESI pos	11.93
	PE(24:0/18:2)	826.6331	ESI pos	13.52
	PE(26:0/18:2)	854.6644	ESI pos	14.43
	PE(28:0/18:2)	882.6957	ESI pos	14.97
PGs	PG(19:1)	561.2547	ESI neg	12.71
	PG(16:0/19:1)	747.5461	ESI neg	13.71

	PG(20:0/22:4)	859.5766	ESI neg	12.60
	PG(24:0/18:2)	857.6277	ESI neg	16.32
	PG(26:0/16:0)	861.6590	ESI neg	18.52
PIs	PI(14:1)	541.2464	ESI neg	10.21
	PI(15:1)	556.2432	ESI neg	10.35
	PI(18:0/22:6)	875.5491	ESI neg	12.76
	PI(18:1/18:1)	861.5499	ESI neg	13.26
	PI(18:3/18:2)	855.5029	ESI neg	10.33
	PI(20:0/18:3)	897.5873	ESI neg	4.30
	PI(24:0/18:2)	945.6438	ESI neg	16.32
PSs	PS(16:0)	502.2589	ESI neg	3.45
	PS(20:0/21:0)	884.6331	ESI neg	10.89
Cers	Cer(d18:1/24:0)	764.5719	ESI neg	13.61
	Cer(d18:0/20:0)	596.5984	ESI neg	13.05
SMs	SM(d18:1/22:1)	805.6177	ESI neg	10.91
	SM(d18:2/18:1)	691.5624	ESI pos	6.42
TGs	TG(14:0/16:0/20:0)	852.8019	ESI pos	20.39
	TG(14:1/20:0/22:0)	958.8808	ESI pos	19.17
	TG(14:1/20:0/22:1)	956.8920	ESI pos	19.10
	TG(14:1/20:0/22:2)	954.8978	ESI pos	19.02
	TG(15:0/18:1/20:1)	890.8183	ESI pos	20.15
	TG(15:0/18:2/20:3)	884.7708	ESI pos	17.26
	TG(16:0/18:0/20:0)	908.8399	ESI pos	20.76
	TG(16:0/18:1/20:0)	906.8496	ESI pos	20.74
	TG(16:0/18:1/20:1)	904.8337	ESI pos	20.36
	TG(16:1/18:2/20:3)	896.7713	ESI pos	16.71
	TG(17:2/20:2/21:0)	956.8659	ESI pos	20.43
	TG(17:2/20:2/22:0)	970.8810	ESI pos	20.64
	TG(17:2/20:2/22:1)	968.8838	ESI pos	20.68
	TG(17:2/21:0/22:2)	984.8973	ESI pos	20.79
	TG(18:1/20:0/22:2)	986.9131	ESI pos	21.06
	TG(18:2/19:0/20:2)	960.8056	ESI pos	19.20
	TG(18:2/19:0/20:4)	962.8209	ESI pos	19.25
	TG(18:3/19:0/20:3)	962.8276	ESI pos	19.23
	TG(18:4/19:0/20:2)	962.8070	ESI pos	19.26
	TG(18:4/19:0/22:0)	985.8877	ESI pos	17.90
	TG(20:0/18:1/16:0)	906.8499	ESI pos	20.72
	TG(20:0/20:1/22:0)	965.8823	ESI pos	22.62
	TG(20:0/20:3/22:0)	961.8819	ESI pos	20.75
	TG(20:0/20:4/22:0)	959.8819	ESI pos	20.68
	TG(24:1/18:2/18:2)	982.8797	ESI pos	25.30
	TG(25:0/18:1/18:1)	1002.9423	ESI pos	25.81
	TG(25:0/18:1/18:2)	1000.9267	ESI pos	25.69
	TG(25:0/18:2/18:2)	998.9110	ESI pos	25.52

	TG(26:0/16:0/18:1)	990.9423	ESI pos	25.90
	TG(28:0/16:0/18:1)	1018.9736	ESI pos	26.09
	TG(28:0/18:1/18:1)	1044.9893	ESI pos	26.03
	TG(28:0/18:1/18:2)	1042.9736	ESI pos	25.87
	TG(30:0/18:1/18:1)	1073.0206	ESI pos	26.20
	TG(30:0/18:1/18:2)	1071.0049	ESI pos	26.03
	TG(30:1/18:1/18:2)	1068.9893	ESI pos	25.80
